# Development and reliability of the coding system evaluating maternal sensitivity to social interactions with 34- to 36-week postmenstrual age preterm infants

**DOI:** 10.3389/fpsyt.2022.938482

**Published:** 2022-10-05

**Authors:** Laure Boissel, Emeric Pinchaux, Marine Guilé, Pascal Corde, Cécile Crovetto, Momar Diouf, Charlotte Mariana, Jonathan Meynier, Carl Picard, Daphné Scoury, David Cohen, Xavier Benarous, Sylvie Viaux-Savelon, Jean-Marc Guilé

**Affiliations:** ^1^Department of Psychiatry, Université Picardie Jules Verne, Amiens, France; ^2^Department of Medicine, Université Libre de Bruxelles, Brussels, Belgium; ^3^Department of Medicine, Université Paris Descartes, Paris, France; ^4^Child and Adolescent Psychiatry Services, University Hospital Centre (CHU), Amiens, France; ^5^Child and Adolescent Psychiatry Services, APHP-GHPS, Sorbonne Université, Paris, France; ^6^Child and Adolescent Psychiatry Services, Hospices civils de Lyon, Hôpital de la Croix Rousse, Université Lyon 1, Lyon, France; ^7^Department of Psychiatry, McGill University, Montreal, QC, Canada

**Keywords:** preterm, maternal sensitivity, infant, mother-child relations, videorecording

## Abstract

Maternal sensitivity (MS), the ability to perceive and synchronously respond to the social signals (SSs), is affected by prematurity. The development of early supportive psychotherapy to foster MS, before discharge of the infant from the neonatal intensive care unit (NICU) is a major challenge in the prevention of subsequent developmental and mental disorders in the child. There are currently no reliable methods for evaluating MS to social interactions with very to moderate preterm infants. We investigated the reliability of a newly developed procedure for assessing MS in interactions between the mother and her 34- to 36-week postmenstrual age (PMA) preterm infant: the Preterm Infant Coding System for Maternal Sensitivity (PRICOSMAS).

**Method:**

This study encompassed three steps: testing of the capacity to videorecord SSs in very to moderate preterm infants, selection, by an expert committee, of the recordable and relevant SSs, and investigation of the internal consistency and interrater reliability. The synchronicity between infant and mother's SSs was determined on a 1 s period basis, using ELAN software. Preterm infants born after 25-weeks gestational age (GA) were included while being between 34- and 36-weeks PMA. A perinatal risk inventory score > 10 for the infant precluded from inclusion. Interrater reliabilities were assessed independently by two raters blind to the clinical situation of the mother and infant.

**Results:**

The resulting PRICOSMAS encompassed two four-item SS sections, one covering the preterm infant's SSs and the other, the mother's SSs. Reliability was assessed on a sample of 26 videorecorded observations for 13 mother-preterm infant dyads. Infants' mean age at birth was 30.4 ± 3.1-weeks GA (range: 26.4–35) and PMA at the time of the test was 34.7-weeks (±0.8). Internal consistency ranged from 0.81 to 0.89. Interrater reliability ranged from substantial to almost perfect (0.73–0.88).

**Conclusion:**

This study shows that the infants' SSs and MS can be reliably scored in preterm infants as young as 34- to 36-weeks PMA. Our findings suggest that the PRICOSMAS is sufficiently reliable for use, including in NICU, by healthcare professionals or researchers for coding early parent-infant interactions with 34- to 36-week PMA preterm infants.

## Introduction

Every year, 15 million babies are born preterm and survive, thanks to progress in the support of high-risk pregnancies over the last 10 years. However, these preterm infants have a higher risk than full-term infants not only of cerebral vascular accidents, but also of attachment disorders, delays in cognitive development, and psychological disorders, including emotional regulation disorders (1, 2). These disorders can persist beyond childhood, into adolescence and adulthood (3). Nevertheless, the developmental trajectories of these infants are highly heterogeneous (4, 5). The mechanisms linking preterm birth to these disorders involve multiple epigenetic, developmental, and environmental factors (6). One of these factors in particular, the quality of parenting and early interactions between infants and their parents, makes an essential contribution (7, 8). Our study fits into the wider context of perinatal research on the impact of early interactions on the development of preterm infants and interventions to support early parent-preterm infant interactions.

Early infant-parent interactions have several components, including parental sensitivity. Parental sensitivity is defined as the ability to perceive and discriminate between the child's social signals (SSs) so as to respond adequately to the child's needs (5, 9). It is the cornerstone of early parent-child interactions and facilitates construction of the child's attachment patterns. Perinatal stress and maternal psychological state influence maternal sensitivity (MS), altering the ability to detect and interpret the child's social signals, and indirectly influencing the quality of early parent-child interactions (8). Mothers who experienced a preterm birth are at higher risk of developing post-traumatic stress disorder (10) (REF). For this reason, many parent-infant interventions, including the reading of stories or fairytales, have been developed in recent decades, to support the development of parental sensitivity and early parent-child interactions (11). Such therapy, especially the reading of stories to the mother-infant dyad, aims to promote MS to the infant's SSs, to prevent the subsequent occurrence of cognitive and psychological development disorders in the infant. The precocity of therapy, with implementation before discharge from the neonatal intensive care unit (NICU), is a key dimension of efficacy (12, 13).

There is a need to assess the impact of such therapies on MS, right from their implementation in the NICU. However, most studies to date have evaluated the impact of interventions much later, when the infant reaches the age of 3 months, rather than in the first few weeks of life, before discharge from the NICU. Furthermore, no such studies focusing on shared reading in preterm infants have been published (14).

Several scales, the psychometric properties of which have been assessed in reliability studies, are used to assess MS in observations of early mother-infant interactions: the Dyadic Mini Code (DMC) developed for preterm infants (15), three scales developed for infants: the Emotional Availability Scales (EAS) (16), the Welch Emotional Connection Screen (WECS) (17) and the Coding of Interactive Behavior-Newborn (CIB) scale developed for infants (18), and the Attachment During Stress Scale (ADS) developed for children aged up to 18 months (19).

The ADS has been used to assess infant behavior during stressful events in very preterm infants (20). However, in this study, several items of the scale were not observable at this age. The item “reaching toward and touching mother” was the least frequently observed.

The CIB was developed to assess parent-infant interactions in feeding situations (18). It includes three behavior categories for each partner involved in the interaction: gaze, touch patterns, and mutual postural adaptation (proximity). It has been used to assess the behavior of moderate to late preterm infants (21). However, in this study, the only item observed for infants was infant feeding performance (feeding-related mouth movements).

MS has been assessed in late preterm infants using the WECS at 4-month corrected age (17). Of note, MS was previously assessed with the Ainsworth System for Rating Maternal Caregiving Behavior when the infants reached 36-week GA.

The EAS were used in preterm infants during their postnatal home visit (22), putting emphasis on the role of mutual touch in mother-infant interactions. However, not all the scale items could be observable in preterms at 34-week PMA.

The DMC has been used to assess mother-infant mutual dyadic responsiveness in moderate to late preterm infants (23). The DMC encompasses six domains of the mother-infant interaction: mutual attention, positive affect, mutual turn-taking, maternal pauses, infant clarity of cues, and MS to cues and responsiveness. The DMC was coded during a play session involving the mother and the infant at a corrected age (CA) of 6-weeks. This instrument is largely based on the imitation competencies already developed by this stage. Based on this first encouraging result, another study used the DMC to assess MS in mother-child interactions in very preterm infants before discharge from the NICU (24). The authors concluded that the instrument was not sufficiently sensitive. More specifically, certain infant items, such as “mutual attention” or “clarity of cues” (scoring based on how clearly the infants indicated, through their behavior, a desire for the stimulation to continue or cease), require social cognition skills of a much higher level than is usually acquired by preterm infants before discharge from the NICU.

We compensated for the lack of a suitable instrument for very preterm infants, by developing a new coding system evaluating MS to SSs in preterm infants admitted to the NICU. Here, we report on the development and reliability of the Preterm Infant Coding System for Maternal Sensitivity (PRICOSMAS). This instrument is designed for use by all professionals working in the NICU, whether clinicians or researchers, for assessments of the impact of therapeutic interventions on MS to very preterm infants' SSs.

## Methods

### Setting

This reliability study is part of a larger study exploring the cognitive and physiological synchronies at work in early interactions between preterm infants and their mothers during a parent-infant shared book-reading session (SYNCHROPREMA ClinicalTrials.gov Identifier: NCT03114644) conducted by the Infant Psychiatry Liaison team of the child and adolescent psychopathology department with parent-preterm infant dyads admitted to the NICU at Amiens university hospital (France). The study was approved by the regional research Ethics Committee (*Comité pour la Protection des Personnes Nord-Ouest*) in accordance with French Bioethics regulations. Written informed consent for participation in this study was provided by the participants' legal guardian for the infant and by the mother for herself. Study data are presented according to the GRRAS guidelines (25).

We identified all relevant studies on the reliability of the aforementioned scales. The study was then performed in three steps: (1) testing of the capacity to videorecord SSs in very to moderate preterm infants, at a PMA of 34- to 36-weeks, (2) selection, by an expert committee, of the relevant infant and maternal SSs for inclusion in the PRICOSMAS from the recordable SSs, and (3) investigation of the internal consistency and interrater reliability of the PRICOSMAS.

### Development of the instrument

In keeping with previous studies using the three available instruments in preterm infants and analyses performed on the synchrony of mother-full-term infant interactions conducted by Chetouani and Cohen (26, 27), we drafted a first list of SSs to be videorecorded and scored during a shared book-reading session with mothers and their preterm infants. This list included the same items for both the mother and preterm infant: vocalization, smiling, gazing, touching, imitation, and postural movement. The items were mutually exclusive and scored as present/absent, allowing the frequency of occurrence to be calculated.

A pilot coding study was conducted on six reading sessions with mother-preterm infant dyads videorecorded before discharge from the NICU (28). The recording, detection and scoring of maternal SSs was feasible. By contrast, certain SSs, such as postural movement and imitation, were not reliably detectable and scorable in very to moderate preterm infants, due to the developmental gap between preterm and full-term infants. Very preterm infants may develop similar imitation capacities to full-term infants, but these competencies are detectable only after discharge from the NICU, when the infant has a PMA of at least 40-weeks (29). The vast majority of studies evaluating the imitation capacity of preterm infants performed observations at a corrected age of 6 or 12 months (30). In addition, the setting of our observations required the preterm infant to be held in the arms of the mother, making it difficult to observe the nascent motor skills of the premature infant fully. Finally, at this age, we observed mouth movements, rather than genuine smiling, as such. The SSs listed in the scales available for assessing the interactions of full-term infants therefore had to be adapted for the observation of very to moderate preterm infants.

### Pretest of maternal sensitivity

MS is defined as the maternal response to the social cues from the child, within a given time interval after the appearance of the social behavior cue in the child. According to the methodology developed by the team of M Chetouani and D Cohen (26, 27, 31), we fixed the observation timeframe to 2 s, making it possible to detect maternal responses occurring within 1 s of the infant's SS. We used ELAN software version 5.9 (Max Planck Institute for Psycholinguistics^®^), which is widely used in the analysis of early parent-child interactions (13, 32). This software can be used for the manual annotation of video or audio resources and the construction of two time series, corresponding to the behavior of the mother and that of the child. The two series are rated independently. The software then compares the two behavioral time series. The behaviors to be annotated are defined in advance by the researchers. For this second pilot study, we used a broader list of SSs for the mother and preterm infant. The ELAN software allowed for triple annotation: occurrence, duration, and timing of SSs.

We conducted a pilot reliability study on a new sample of ten videos recorded during a shared book-reading session with mothers and preterm infants. Two trained raters (LB, M.D). and MG, an MSc. Student independently scored the videos with ELAN software: occurrences, duration, and timing of maternal and preterm infant SSs. Interrater reliability (Kappa coefficient) for the SSs of the preterm infants ranged from 0.66 to 0.86. Based on these coefficients, we retained the following preterm infant SS items: eye opening/closure, changes in gaze orientation, mouth opening/closure and vocalization. We chose to annotate all mouth opening/closure movements with ELAN rather than specifying and labeling certain mouth movements as smiles. This choice is consistent with that made by Palazzi et al. (13) in a study on a sample of preterm infants with a mean PMA of 36.75-weeks at the time of testing. In that study, opening or closure of the eyes or mouth was annotated with ELAN as a sign of the engagement of the preterm infant in music therapy sessions. We chose not to consider the body movements of the infants, who were placed in the arms of their mothers during the reading session, to ensure their comfort, as this limited infant movements. Finally, arm movements are rare in preterm infants with a PMA of 34- to 36-weeks, due to the effects of gravity.

Maternal behaviors were annotated with ELAN within the timeframe for observation following infant behavior (1–2 s). Interrater reliability (Kappa coefficient) for the mothers' SSs ranged from 0.56 to 0.92. Based on these results and the items generally retained in the cited instruments for evaluating mother-child interactions, we retained the following items as maternal SSs indicative of MS to the SSs of preterm infants: maternal touching of the child, smiling at the child, facial expressions (other than smiles) directed at the child, and maternal vocalization.

### Face validity and final selection of items

The precise definition of the maternal and preterm infant items was discussed until a consensus was reached. These items were then validated by a committee of experts composed of Professor David Cohen, Professor Guilé, and Doctor Sylvie Viaux (Table 1). This expert committee also decided on the time interval for which maternal behavior could be considered to be a response to infant behavior, and, therefore, to constitute a marker of MS. Based on previous studies on turn-taking in early parent-child interactions (27, 33), and the feasibility and reliability of the detection of maternal SSs and determination of their timing during our pre-test, the committee decided to consider maternal SSs occurring within 1 s of a preterm infant SS as responses to the preterm infant's SS. The SSs were annotated as soon as they appeared. It was also decided that the time to the response should be measured from the initiation of the infant's SS. Indeed, preterm infants with a PMA of 34- to 36-weeks act slowly, and maternal responses may be initiated before the end of the movement, to support the action initiated by the preterm infant.

**Table 1:** List of social signals (SSs) included in the Preterm Infant Coding System for Maternal Sensitivity (PRICOSMAS).

**Social signal (SS) items**	**Description**
**Preterm infant's SSs directed toward the mother**	
Eye opening/closure	Change in the position of the eyelids, passage from opening to closing the eyelids and vice versa.
Change in gaze orientation	With the eyes open, a change in the orientation of the eye, regardless of direction.
Mouth opening/closure	Movement of the mouth, opening or closing of the mouth. May include lip lifts and smiles.
Vocalizations	All vocalizations, screaming or crying.
**Mother's SSs directed toward the preterm infant**	
Touching	Touching of the infant by the mother. Can included caressing.
Smiling	Maternal smile directed at the infant.
Facial expressions	Any movement of the face or eyebrows for which the mother's head is turned toward the child. Smiles and rotations of the head are not scored in this category.
Vocalizations	Words, interjections, or exclamations directed at the child.

### Reliability study

**Figure 1:**
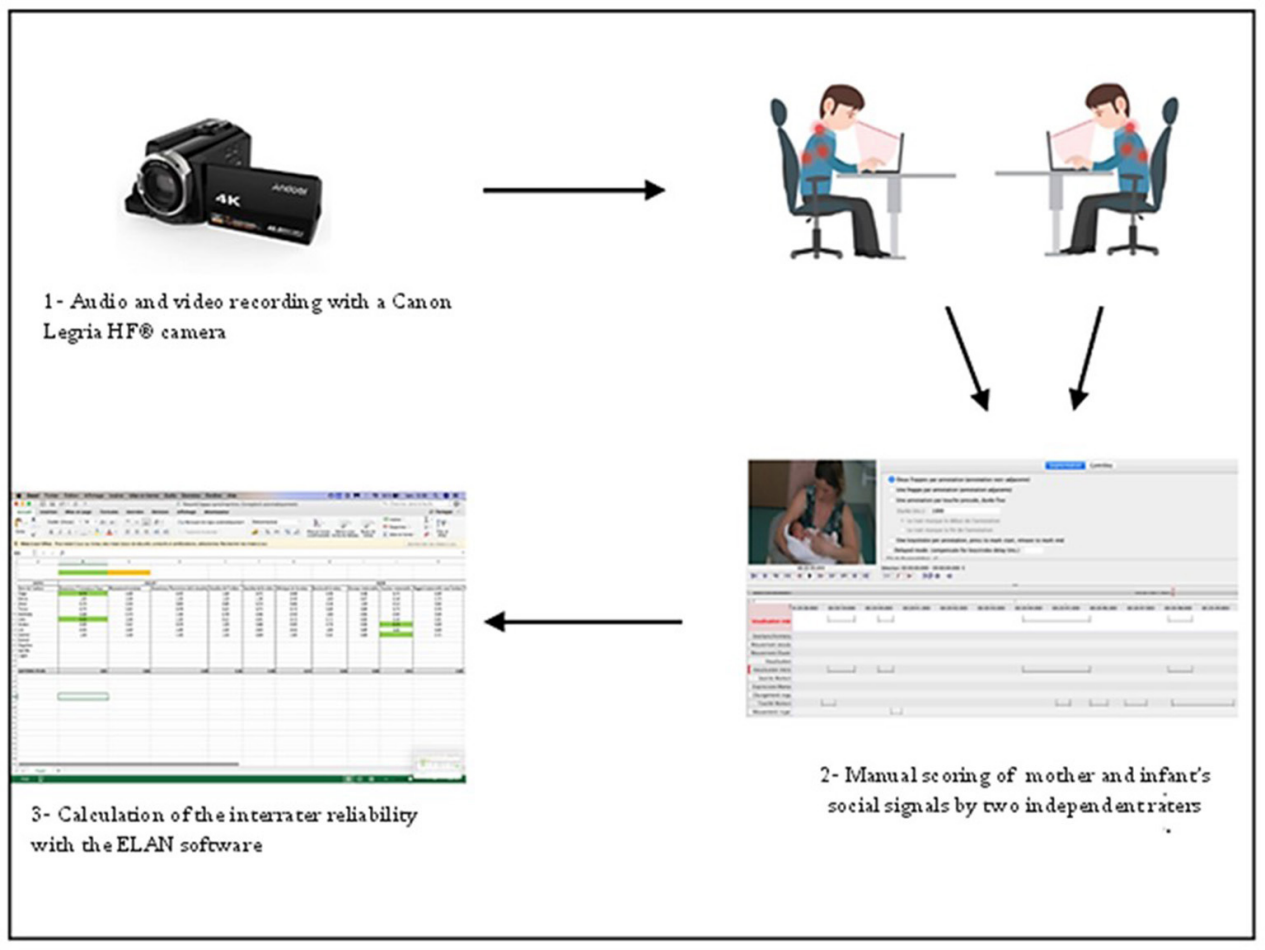
Reliability study procedure.

The study took place in the same setting as the pretest studies. Videorecordings and PRICOSMAS scoring took place during (Figure 1) parent-infant shared book-reading sessions, which are commonly provided by the Infant Psychiatry Liaison team as a means of supportive psychotherapy for parents with preterm infants in the NICU (14).

This study targeted extremely to moderate preterm infants admitted to a university teaching hospital NICU and their mothers. The infant participants were born at 26- to 36-weeks of gestational age (GA). The exclusion criteria were as follows:

– For the mother: a psychiatric disorder including a substance-related and addictive disorder, guardianship or curatorship, or a lack of understanding of the French language.
– For the child: being a twin, a genetic disease, a progressive neurological disease, or a Perinatal Risk Inventory (PRI) score > 10 (34).

### Sample size calculation and procedure

The reliability study investigated the use of PRICOSMAS to assess MS during interactions between mothers and their preterm infants during videorecorded shared book-reading sessions. For a Cohen's Kappa coefficient of 0.7, the power analysis showed that 26 observations would be required to show that the lower limit of the 95% confidence interval was not <0.4, given a frequency of the observed feature of 50%.

The participating mother-preterm infant dyads were admitted to the NICU between May 2018 and January 2020. The Infant Psychiatry Liaison team was systematically informed of any new potentially eligible dyads during hospitalization of the infant in the intensive care unit. The inclusion and exclusion criteria were assessed by the psychiatrist responsible for inclusions, based on an analysis of the child's medical file and an interview with the mother. The PRI score was calculated by the referring pediatrician from: (1) APGAR score, (2) the child's PMA at birth, (3) the child's weight at birth, and (4) the child's head circumference at birth. A threshold of 10 (range: 0–51) was used, based on studies showing a significant risk of developing developmental disabilities above this threshold (34).

As maternal mood can influence the quality of early mother-infant interactions, the participating mothers were asked to complete two self-administered questionnaires: (1) the Beck Depression Inventory (BDI) (35) and (2) the State-Trait Anxiety Inventory (STAI A and B) (36) to assess the severity of depressive and anxious symptoms, respectively.

### Procedure

We aimed to develop an assessment instrument for use by clinicians and researchers with a health science (nursing, SLP, OT, medicine), SW, or psychology bachelor's degree, working with preterm infants and their parents. This instrument should be useable both in constrained environments, such as a hospital NICU, and during home visits. All the raters involved in this study were MSc students.

Videorecording was performed throughout the parent-infant shared book-reading session during the preterm infant's stay in the NICU. The session took place in the NICU room as follows: the mother and the preterm infant were initially left alone in the room for 5 min. The professional from the Infant Psychiatry Liaison team then arrived, introduced herself, and began the session, reading a fairytale or story chosen in advance by the mother. The mother and preterm infant were then left alone again for another 5 min. The sessions were conducted away from care activities and approximately half an hour before breast or bottle feeding, a period during which preterm infants are generally awake (37).

During the reading session, the preterm infant was expected to be in a state of attentive alertness, with the eyes open [stage 3 on the Brazelton scale (38)]. The mother was asked to interact as usual with her preterm infant before, during, and after each reading. The videos were recorded with a “Canon Legria HF r606” camera and stored in MP4 format.

Videoscoring for the PRICOSMAS, with ELAN software, was based on the recordings made before and after the reading of the story. The videos were rated by two health science MSc students not involved in the pretest studies or construction of the instrument (39, 40). They were trained for half a day by the research team, with a video not included in the study sample. Each PRICOSMAS item (Table 1) was scored as many times as the SS appeared on the video and was coded according to a binary rating system (present/absent). Each rater rated the videos independently, with the PRICOSMAS, blind to clinical information from the participants or the care staff. The two researchers were not together when they rated the videos and could not discuss their ratings. They did not discuss any clinical information and they were both aware that their ratings would be compared.

### Statistical analysis

Two types of reliability analysis were conducted with IBM SPSS^®^ Statistics version 24: (1) internal consistency, based on the calculation of Cronbach's alpha coefficient for the preterm infant SS and mother SS sections of the instrument, and (2) interrater reliability, based on calculation of the Kappa coefficient for each of the items. We used the Landis and Koch scale to assess the significance of inter-rater agreement (41).

## Results

### Sample characteristics

The observations consisted of two videos for each of the 13 mother-preterm infant dyads, giving a total of 26 video observations. Each video was rated by the two raters. APGAR score was between 8 and 10 ± 1 at birth for the 13 preterm infants. The infants studied were born at a mean of 30.4 ± 3.1-weeks of gestation age (GA), ranging from 26-weeks and 4 days to 35-weeks GA. Mean head circumference was 31.26 ± 5.78 cm and mean weight was 1.77 ± 0.35 kg at birth. The preterm infant's PMA at the time of testing was 34.7-weeks (±0.8).

The 13 mothers who participated in our study were between 19 and 38 years of age, with a median age of 29 ± 5.8 years; 69% of the mothers were primiparous and 8% were single. The level of education of the mothers was distributed as follows: 77% university, 15% secondary school, and 8% elementary school.

The mean scores for the depression and anxiety tests were 30 ± 10 for STAI A, 38 ± 8.2 for STAI B and 4 ± 2.7 for the BDI. The mothers participating in the study therefore presented no clinically observable or self-assessed signs of depression.

### Internal consistency

Following the pretest studies and the process of confirmation by the expert committee, the resulting PRICOSMAS instrument encompassed two four-item SS sections, one covering the preterm infant's SSs and the other, the mother's SSs. Item-total correlations for the infant section ranged between 0.62 and 0.74. Item-total correlations for the maternal section ranged between 0.62 and 0.86. The Cronbach alpha coefficients were 0.81, 0.86, and 0.89 for the infant section, the maternal section, and the total scale, respectively. Thus, the development of the PRICOSMAS and the subsequent pretest studies resulted in an instrument with excellent internal consistency.

### Interrater reliability

The kappa coefficients are presented in Table 2. According to the Landis and Koch classification (41), the interrater reliability of the PRICOSMAS was in the substantial to almost perfect range.

**Table 2:** Inter-rater reliability.

**Social signal (SS) items**	**Kappa coefficient**	**95% ICC**
**Preterm infant's SSs directed toward the mother**	
Eye opening/closure	0.79	0.68–0.90
Change in gaze orientation	0.82	0.70–0.94
Mouth opening/closure	0.88	0.81–0.95
Vocalizations	0.86	0.76–0.96
**Mother's SSs directed toward the preterm infant**	
Touching	0.80	0.73–0.87
Smiling	0.87	0.79–0.94
Facial expressions	0.73	0.62–0.83
Vocalizations	0.81	0.70–0.91

ICC, intraclass correlation coefficient.

## Discussion

Collaborative work between specialists, clinicians, and researchers over the last 4 years on early interactions between parents and preterm infants led to the development of an instrument for rating the SSs exchanged between mothers and their preterm infants. This instrument has excellent psychometrics and can be used by professionals educated to the bachelor's degree level.

The maternal SS items are similar to those used in scales for analyzing interactions with infants born at term. Conversely, the SSs proposed in the PRICOSMAS for the observation of preterm infants are different from those for full-term infants, as they consider the motor and communication skills actually demonstrated by the preterm infant, particularly for very to moderate preterm infants.

We found that the reliable detection of signals emitted by preterm infants with a PMA as low as 34-weeks was feasible. With ELAN software, the PRICOSMAS can be used to rate the frequency, duration, and timing of the SSs listed in our instrument, and those of the maternal response SSs. If the mother's SS occurs within 1 s of the preterm infant's SS, it is interpreted, in our theoretical model, as a sign of MS. The succession of SSs emitted by the preterm infant and the mother, thus, progressively constitutes a dynamic synchrony with a communicational and pre-linguistic valence.

The study by Hane et al. (17) used the Welch Emotional Connection Screen (WECS) at 4-month CA. The study by Mantis and Stack (22) used the Emotional Availability Scales (EAS) during the 4–5 month post-natal home visit. Apart the Dyadic Mini Code (DMC), none of the aforementioned available instruments was used to assess MS with preterm infants younger than 37-week PMA/GA before discharge from the NICU (24). Rating is easier with our scale than with the DMC, the only other instrument originally constructed for use with preterm infants, because it does not assume that the SSs observed are of communicative value. The study by Eutrope et al. (24) showed that the DMC was unsuitable for observations of early interactions with very preterm infants. Unlike the DMC, our instrument distinguishes between the time to detect an SS and the time taken by the mother to evaluate the SS and display MS, by scoring the occurrence and timing of maternal SSs within 1 s of the SS of the infant.

Unlike the instruments developed for use with full-term infants and subsequently used with preterm infants, our instrument was specifically developed to detect the SSs of preterm infants and holds characteristics that are consistent with the observations of researchers who have used scales developed for full-term infants on preterm infants. Our instrument does not score the motor movements of preterm infants. Indeed, as noted by the researchers who used the Attachment During Stress Scale (ADS) (20) and observed in the pretest of our instrument, preterm infants rarely move their upper limbs, and such movement is hampered by the “kangaroo” position of the preterm infants against their mothers. The rarity of such signals precludes reliable scoring, as the scoring of an infrequent item increases the probability of inter-rater disagreement.

We chose not to attribute *a priori* communicational or intentional value to the SSs or behaviors of the preterm infant. This was based on our theoretical model, in which the engagement in a dynamic mother–preterm infant synchrony (26, 31) provides communicational value to the SS signals observed in preterm infants. This is the reason for which we labeled one of the items “mouth opening/closure” rather than “smile,” even though those close to the baby would interpret this mouth movement as a smile. In this sense, this item has a definition very similar to that of the “smile” item in the work of Palazzi et al. (13). Palazzi et al. considered a “smile” item to be a movement of the preterm infant's upper lip. It should also be noted that this movement, also observed during feeding, was the only item retained from the CIB for the observation of preterm infant-parent interactions in the study by Silberstein et al. (21).

Our work has implications for research and clinical practice. The PRICOSMAS can be used for video rating of MS to SSs emitted by preterm infants, both in the context of a study (as here, in a larger project evaluating the effect of reading stories), and in the evaluation of clinical practices. There is currently no instrument adapted to the psychomotor development of preterm infants suitable for use in the analysis of MS. However, this is a crucial issue in clinical practice and the prevention of early interaction disorders. MS is the cornerstone of the construction of early interactions and current support therapies aiming principally to strengthen MS, particularly in situations in which neonatal stress reduces this maternal capacity.

Our study had several limitations. The size of the sample and the relative GA heterogeneity of gestational age, together with recruitment at a single site, limit the generalizability of the results. In addition, PRICOSMAS has been developed in the context of NICU interventions, more specifically the parent-preterm infant shared reading therapy. The question remains about the applicability of our procedure to other NICU interventions, naturalistic interventions, and physiological research (42).

However, our study shows that infant SSs and MS can be reliably scored in interactions with preterm infants with a PMA as low as 34- to 36-weeks. The PRICOSMAS appears to be a promising instrument for use by healthcare professionals or researchers for coding early parent-infant interactions with preterm infants with a PMA of 34- to 36-weeks.

## Data availability statement

The raw data supporting the conclusions of this article will be made available by the authors, without undue reservation.

## Ethics statement

The studies involving human participants were reviewed and approved by Comité de Protection des Personnes Nord-Ouest. Written informed consent to participate in this study was provided by the participants' legal guardian/next of kin.

## Author contributions

LB, PC, MD, DS, DC, XB, SV-S, and J-MG contributed to conception and design of the study. LB, EP, MG, PC, CM, DS, and J-MG contributed to recruiting and data collection. MD, JM, and CP contributed to data management and monitoring. LB, EP, MG, MD, JM, CP, and J-MG contributed to the statistical analyses. LB, SV-S, and J-MG wrote the first draft of the manuscript. All authors contributed to data analyses, manuscript revision, read, and approved the submitted version.

## Conflict of interest

The authors declare that the research was conducted in the absence of any commercial or financial relationships that could be construed as a potential conflict of interest.

## Publisher's note

All claims expressed in this article are solely those of the authors and do not necessarily represent those of their affiliated organizations, or those of the publisher, the editors and the reviewers. Any product that may be evaluated in this article, or claim that may be made by its manufacturer, is not guaranteed or endorsed by the publisher.
